# Progress in Early Detection of HIV in Tajikistan

**DOI:** 10.3390/v16071010

**Published:** 2024-06-23

**Authors:** Kamiar Alaei, Brian Kwan, Hamid R. Torabzadeh, Adebimpe O. Akinwalere, Sattorov S. Saydamirovich, Gafur Mohsinzoda, Arash Alaei

**Affiliations:** 1 Health Science Department, College of Health and Human Services, California State University Long Beach, Long Beach, CA 90840, USA; kamiar.alaei@csulb.edu (K.A.); brian.kwan@csulb.edu (B.K.); adebimpe.akinwalere01@student.csulb.edu (A.O.A.); 2 Institute for International Health and Education, Albany, NY 12207, USA; 3 School of Public Health, Brown University, Providence, RI 02912, USA; 4 Tajikistan Ministry of Health and Social Protection of Population Republican AIDS Center, Dushanbe 734000, Tajikistan; 5 Tajikistan Ministry of Health and Social Protection of Population, Dushanbe 734000, Tajikistan

**Keywords:** HIV detection, HIV testing, Tajikistan, Central Asia, regional disparities, rural areas, gender disparities, age, COVID-19, case study

## Abstract

HIV early detection (CD4 counts ≥350 cells/μL) is correlated with higher life expectancy among people living with HIV (PLHIV). Several factors, including physical, cultural, structural, and financial barriers, may limit early detection of HIV. This is a first-of-its-kind study on population-level differences in early detection of HIV across time within Tajikistan and any country in the Central Asia region. Utilizing the Tajikistan Ministry of Health’s national HIV data (N = 10,700) spanning 2010 to 2023, we developed median regression models with the median CD4 cell count as the outcome and with the following predictors: time (years), region, age, gender, and area (urban/rural status). Individuals younger than 19 years old were detected early for HIV, whereas those older than 39 years were detected late. Females were detected earlier compared to their male counterparts regardless of region of residence. Rural populations were detected earlier in most years compared to their urban counterparts. The COVID-19 pandemic accelerated HIV early detection in 2021 but most regions have returned to near pre-pandemic levels of detection in 2022 and 2023. There were differences identified among different demographic and geographic groups which warrant further attention.

## 1. Introduction

Antiretroviral therapy (ART) has been proven as the most effective intervention to reduce HIV-associated morbidity and mortality among people living with HIV (PLHIV) and prevent subsequent transmission of the virus [1,2]. Early detection is a critical public health intervention to improve treatment outcomes of ART which are significantly impacted by the timeliness of treatment initiation [3,4,5]. The CD4 cell count is the WHO-recommended test to determine the progression of HIV disease and the detection stage [6]. The normal range of the CD4 count is from 500 to 1500 cells/μL of blood while someone with a CD4 count below 200 is described as having an advanced HIV disease (AHD) [6]. Early detection is defined as CD4 counts ≥350 cells/μL [7,8].

Early detection CD4 counts are associated with life expectancy among PLHIV similar to that of the general population [9,10,11,12]. Late HIV detection and diagnosis reduces life expectancy [13], increases the frequency of HIV-related comorbidities [14,15] and is associated with higher health care costs [16]. Further literature in a cohort-based study has confirmed the most significant independent risk factor for incomplete immune recovery is a lower CD4 cell count at ART initiation [17]. Late detection can have adverse effects on the effectiveness of ART on viral suppression. In Eastern Europe and Central Asia, in 2020, 54% of new HIV diagnoses were detected at the late stage (CD4+ < 350 cells), a 10% increase from 2018 [18].

Globally, several physical, cultural, structural, and financial barriers to HIV early detection among specific populations have been identified. There are age disparities with a lower uptake of HIV testing among university students and young people due to limited education on HIV and community engagement [19,20]. There are gender disparities in HIV testing and the receipt of results, HIV stigma, HIV knowledge, and treatment outcomes which differ among men and women [21,22,23,24,25,26]. There are urban and rural disparities in which those who have access to and regularly utilize primary health care services are more likely to be detected and treated for HIV [27,28,29]. Further, urban populations face a greater burden of initial HIV cases due to population density, lower income level, and cultural practices [30,31]. Rural populations experience less access to health care services, more distance traveled for access to testing, and cultural stigma which may in turn prevent early detection and limit the effectiveness of treatment [32,33,34,35,36].

The Republic of Tajikistan (“Tajikistan”) has a growing HIV epidemic with a 2021 prevalence and incidence in adults of around 0.2% and 0.017%, respectively [37]. Challenges for early detection of HIV in Tajikistan include high mobility, stigma and discrimination against those living with HIV, and limitations in funding and public health–healthcare system partnerships [38]. Various cultural and structural barriers may contribute to disparities in early detection of HIV. These barriers include gender disparities across the country and the broader Central Asian region [39]; high stigma following a positive HIV diagnosis [40,41,42]; trickle effects of the war in Ukraine and consequent waves of refugees and migration [43]; economic slowdown during COVID-19 in Tajikistan and globally [44,45,46,47]; and unsustainable international financing [18,48]. Prior literature has also demonstrated gender and regional disparities in linkage to care within Tajikistan, with males, those living in urban areas, and those living in the capital city, Dushanbe, least likely to link with primary HIV care [49,50].

Diagnosing HIV cases at earlier stages can reduce the burden of HIV in Tajikistan and ensure the effectiveness of ART among different populations. Although prior literature has demonstrated various barriers to early detection globally, this paper uses Tajikistan as a case study to elucidate differences in early HIV detection across regions, age, gender, and area (urban/rural status) since 2010. This paper seeks to identify the extent to which demographic and geographic disparities contribute to early HIV detection among the Tajikistan population. Understanding changes over time will contribute to prior literature on early detection barriers which may impact access to testing and linkage to care among specific populations in Tajikistan.

## 2. Materials and Methods

### 2.1. Study Participants

Our study presents an analysis of cross-sectional data from the national registry system operated by the Tajikistan Ministry of Health and Social Protection. The data include all individuals (N = 10,700) who have been diagnosed with HIV by the Tajikistani health system from 1 January 2010 to 30 May 2023. Individuals resided across five regions: Districts of Republican Subordination (DRS), Dushanbe (Tajikistan’s capital city), Gorno-Badakhshan Autonomous Oblast (GBAO), Khatlon, and Sughd. The study consists of individuals across age (years) categories (under 19 y, 19–39 y, over 39 y), identified as female or male gender, and who provided their area of residence (urban/rural status).

CD4 cell counts (cells/μL) were collected at time of diagnosis by Tajikistani healthcare providers and is the WHO-recommended test to determine the progression of HIV disease and the detection stage [6]. As a marker for detecting early to advanced stages of HIV disease, we note that the normal range of the CD4 count is from 500 to 1500 cells/μL of blood while someone with a CD4 count below 200 is described as having an advanced HIV disease (AHD) [6]. Early detection is defined as the first result CD4 counts ≥350 cells/μL [7,8].

### 2.2. Statistical Analysis

We have provided descriptive statistics for demographic characteristics, i.e., count (%) for binary/categorical variables. Our dependent variable is CD4 cell count (cells/μL) at time of detection of HIV. Given the skewness of the distribution of collected CD4 cell count data from the participants, we examined the median CD4 cell count across the years from 2010 to 2023 by the categories of region, age, gender, and area of residence.

We developed median regression models with the median CD4 cell count as the outcome and with the following predictors: time (years), region, age, gender, area of residence. In particular, we fitted these models with two-way interactions for time (years) with region, age, gender, and area of residence, each separately, to elucidate the rates of change in the median CD4 across categories. We conducted Wald tests [51] to test for significant two-way interactions at the 5% level in our models. Median regression is a form of quantile regression that estimates the conditional median for a dependent variable given values from independent variables. The advantages of quantile regression models include not making assumptions on the distribution of the dependent variable and its regression estimates are more robust to outlying observations than ordinary least squares regression. All statistical analysis was conducted using the R programming language [52].

## 3. Results

In Table 1, we provide demographic characteristics of HIV cases in Tajikistan from 2010 to 2023 (N = 10,700). The regions of DRS, Dushanbe, Khatlon, and Sughd each make up at least 20% of the cases in Tajikistan, with Khatlon containing greater than a quarter of the cases alone (28%). The majority of cases belong to the age group of 19–39 years old (62%) and identified as male gender (58%). Additionally, over a quarter of the cases (26%) were aged above 39 years. Approximately 56% of the cases have residence in a rural area, while around 44% of the cases have residence in an urban area.

**Figure 1:**
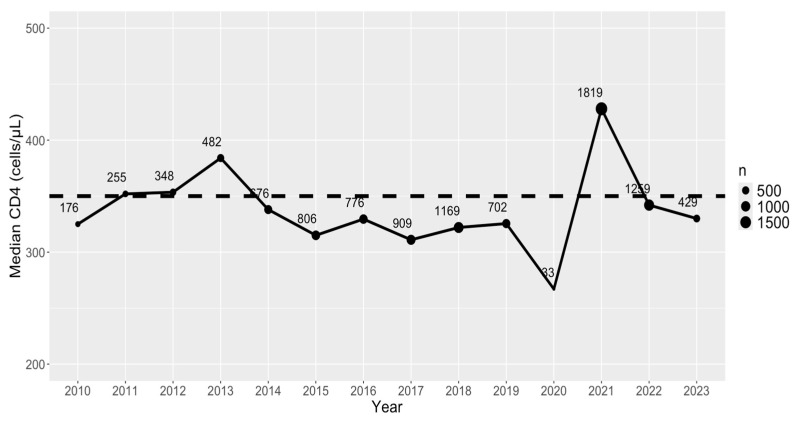
Median first CD4 count per year (2010–2023) and corresponding sample size (n) for CD4 values at each year. Black dashed line is for a CD4 value of 350 cells/μL (early detection).

In Figure 1, we present the median first CD4 count per year from 2010 to 2023. There is an initial increasing trend in median CD4 count values starting from 2010 to 2013, followed by a drop to below the threshold of HIV early detection (CD4 counts ≥350 cells/μL) until 2020, which notably marks the year with the lowest collected number of CD4 samples. Following 2020, the maximum median CD4 count is observed in 2021, coinciding with the largest number of samples. However, the trend subsequently declined thereafter.

**Figure 2:**
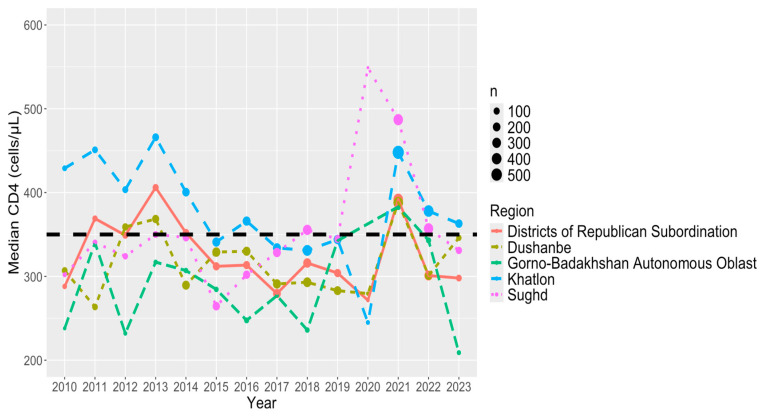
Median first CD4 count per year (2010–2023) by region. Sample size for CD4 values at each year (n). Black dashed line is for a CD4 value of 350 cells/μL (early detection).

In Figure 2, we provide the median first CD4 count per year from 2010 to 2023 by region (DRS, Dushanbe, GBAO, Khatlon, and Sughd). A majority of the median CD4 count across all years is below the threshold of HIV early detection (CD4 counts ≥350 cells/μL) with the exception of Khatlon. Notably, Khatlon has a median first CD4 count consistently greater than 350 cells/μL from 2010–2014 and 2021–2023. All regions experienced a decrease in the median CD4 count at the onset of COVID-19 in 2020 except Sughd. The first COVID-19 case in Tajikistan was detected in April 2020. In the subsequent year 2021, all regions experienced an increase in the median CD4 count except Sughd, with Khalton experiencing the largest increase. However, the trend shifted following the subsequent years, though inconsistently, as it declined across all regions once more.

**Figure 3:**
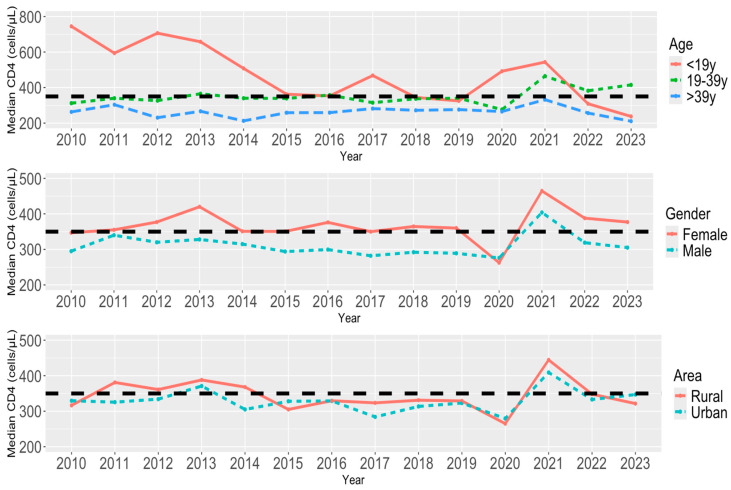
Median first CD4 count per year (2010–2023) by age (**top**), gender (**middle**), and area (**bottom**). Black dashed line is for a CD4 count value of 350 cells/μL (early detection).

In Figure 3, we provide the median CD4 count per year from 2010 to 2023 by our key demographics: age, gender, and area (urban/rural status). When looking at the median CD4 count across all years by age, the median CD4 count for both the age groups of younger than 19 years old and 19–39 years old were consistently greater than that of the group of >39 years old. It appears that individuals younger than 19 years old were more likely to be detected early for HIV (CD4 counts ≥350 cells/μL), whereas those older than 39 years were more prone to late detection (CD4 counts <350 cells/μL). In addition, there was a notable difference in values even just between the <19 years and >39 years groups at the start of the time frame of 2010 to 2015 and at the end of the time frame of 2020–2023, with <19 years old reaching approximately two times the median value of CD4 counts for HIV early detection (700 cells/μL) in the years 2010 and 2012.

When looking by gender, the median CD4 count for females was shown to be greater than that of male individuals across all years with the exception of 2020, which again is limited to a small sample size during that year. Females are more likely to be detected early for HIV (CD4 counts ≥350 cells/μL) compared to males.

When looking by area status (urban/rural) from 2011 to 2014, individuals residing in rural areas exhibited a higher likelihood of early HIV detection compared to those in urban areas. However, the difference in the median CD4 count comparing rural vs. urban areas is less pronounced thereafter. Since 2015, both areas experienced a decreased median CD4 count that was below early detection for HIV (CD4 counts <350 cells/μL), with the exception of the year 2021.

**Figure 4:**
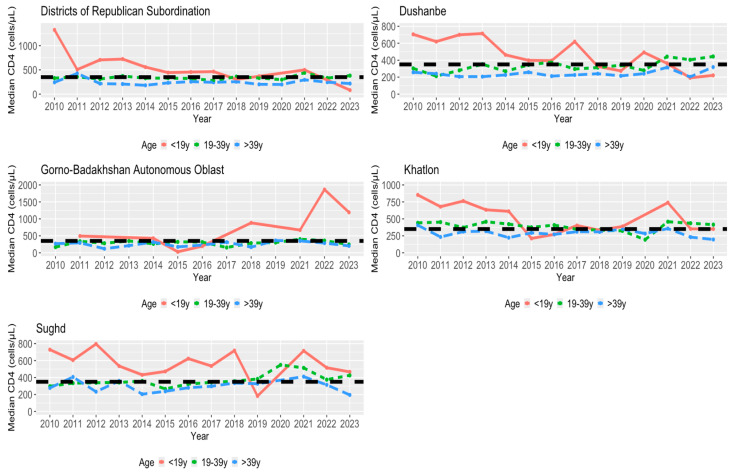
Median first CD4 count for each region per year (2010–2023) by age. Black dashed line is for a CD4 value of 350 cells/μL (early detection).

**Figure 5:**
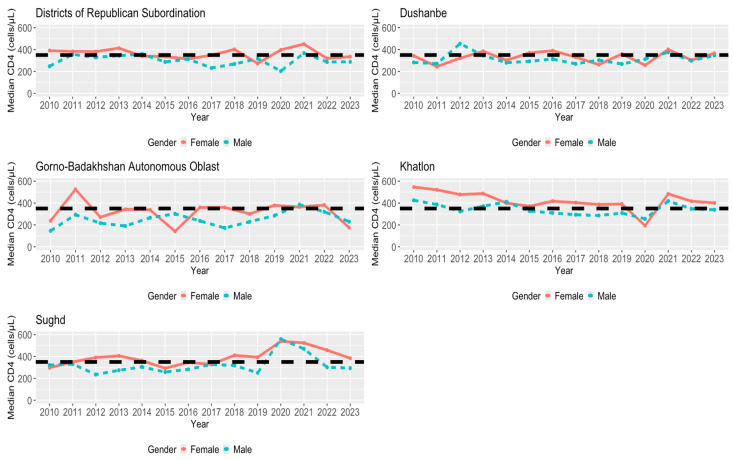
Median first CD4 count for each region per year (2010–2023) by gender. Black dashed line is for a CD4 value of 350 cells/μL (early detection).

**Figure 6:**
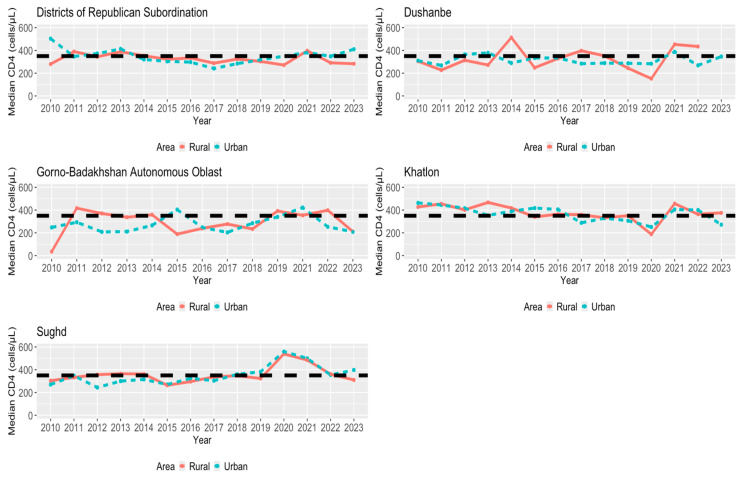
Median first CD4 count for each region per year (2010–2023) by area (urban/rural status). Black dashed line is for a CD4 value of 350 cells/μL (early detection).

In Figure 4, Figure 5 and Figure 6, we examined the median CD4 count per year for each region based on age, gender, and area status (urban/rural status), respectively. In looking at the different age groups for each region, the group of <19 years old individuals had a relatively higher median CD4 count in most of the years for each region. For each region of the <19 age group, DRS was above the HIV early detection threshold for the first half of the time frame, Dushanbe, Khatlon, and Sughd followed similarly to DRS, and GBAO obtained, for the latter half of the time frame, a maximum median CD4 count value at year 2022 that was greater than four times the HIV early detection of 350 cells/μL. Therefore, while the median CD4 count values were above the threshold for DRS, Dushanbe, and Khalton, the trend was declining over time, with an exception in 2020. This trend stood in contrast to GBAO, where the trend was consistently increasing over time, reaching its maximum in 2022. In most instances, females were more likely to be detected early compared to males, regardless of the region. Compared to examining age groups within regions, the trends and patterns for the median CD4 count across years by gender and area status are less pronounced.

In Table 2, we provide three of our fitted median regression models with the median CD4 cell count as the outcome (Models 1–3). Model 1 was selected to analyze time (2010–2023) as a sole predictor for median CD4. We also looked into whether time was a significant predictor for median CD4 by region, age, gender, and area, and found significant differences for time as a predictor by region (Model 2) and by age (Model 3).

Our first model (Model 1) investigates time (years since 2010) as the sole predictor for median CD4 count (*p* < 0.001). In interpreting Model 1, the predicted median CD4 count is 319.11 cells/μL at the baseline year 2010 and the predicted median CD4 count increases by 3.56 cells/μL for a 1-year increase in time since 2010.

Model 2 investigates time (years since 2010) and region of residence as predictors along with the two-way interactions between time and region. Since Dushanbe is the reference group for region as a predictor for Model 2, the predicted median CD4 count at baseline for Dushanbe is 298.03 cells/μL, which indicates that approximately 50% of individuals diagnosed with HIV in Dushanbe were predicted to have a lower CD4 count than 298.03 cells/μL, which is lower than the HIV early detection of 350 cells/μL. In looking at the main effects of region as a predictor (with Dushanbe as the reference), DRS and Khatlon had significant differences in predicted median CD4 count at baseline year 2010 compared to Dushanbe (*p* = 0.046 and *p* < 0.001, respectively), with DRS displaying a higher median CD4 at baseline (Coef. = 41.46) and Sughd displaying a lower median CD4 count at baseline (Coef. = −10.73). Furthermore, for a 1-year increase in time since 2010, the predicted median CD4 count increases by 3.92 cells/μL for Dushanbe and, by conducting a Wald test, we found that there is an overall significant difference in rates of change in median CD4 count across all years 2010–2023 between all five regions of Tajikistan (*p* = 0.006). Here, we can see that the rate of change in median CD4 count for DRS is significantly decreased compared to that of Dushanbe (Coef. = −5.06, *p* = 0.046).

Model 3 investigates time (years since 2010) and age as predictors along with the two-way interactions between time and age. Here, 19–39 y is the reference group for age as a predictor in which the predicted median CD4 count at baseline for 19–39 y is 305.08 cells/μL, which indicates that approximately 50% of individuals diagnosed with HIV in Dushanbe were predicted to have a lower CD4 than 305.08 cells/μL, which is lower than the HIV early detection of 350 cells/μL. In looking at the main effects of age as a predictor (with 19–39 y as the reference), both groups <19 y and >39 y had significantly different predicted median CD4 counts at baseline year 2010 compared to 19–39 y (*p* < 0.001 for both), with <19 y displaying a higher median CD4 at baseline (Coef. = 311.68) and >39 y displaying a lower median CD4 count at baseline (Coef. = −61.24). Furthermore, for a 1-year increase in time since 2010, the predicted median CD4 count increases by 7.75 cells/μL for 19–39 y and, by conducting a Wald test, we found that there is an overall significant difference in rates of change in median CD4 count across all years 2010–2023 between the three age groups (*p* < 0.001). Here, we can see that the rates of change in median CD4 count for both <19 y and >39 y were significantly decreased compared to that of 19–39 y (Coef. = −29.68, *p* < 0.001 for <19 y and Coef. = −4.22, *p* = 0.018 for >39 y).

We also fitted two additional median regression models that investigated time (years since 2010) and gender as predictors along with their two-way interactions and time (years since 2010) and area (urban/rural status) as predictors along with their two-way interactions. Since our interest is in examining whether there are significant rates of change in median CD4 count across all years 2010–2023, we conducted Wald tests in both models and found that the *p*-values for both the interaction between time and gender as well as between time and area were not significant at the 5% level (*p* = 0.489 and *p* = 0.359, respectively).

## 4. Discussion

Nationally, from 2010 to the first half of 2023, although there were sizable improvements in early detection from 2010 to 2013 above the 350 cells/μL global target for early detection, the median CD4 count remained fairly consistent from 2014 to 2019. Prior literature has demonstrated a decline in access to HIV treatment, disruption in care delivery, lower access to ART, and deepening of socioeconomic inequities in many countries as a result of COVID-19 in 2020 [53]. While reduced detection is reflected in the sample size of the 2020 data in this study and Tajikistan suffered from various COVID-19 restrictions which hindered testing during the pandemic, the Tajikistan health system demonstrated improved detection about one year into the pandemic in 2021 with an all-time high median CD4 count of near 450 cells/μL, but this improvement returned to near pre-pandemic detection levels in 2022 and 2023 with a downward trajectory. This demonstrates health system capacity for strong early detection and linkage to care but improvement is necessary to solidify COVID-19-level investments, including increased international aid, use of telehealth, and integration of HIV care into primary care for more sustainable and long-term early detection.

Further, while over the past 14 years, Tajikistan had an overall median CD4 count of 347 cells/μL for a new HIV diagnosis at time of detection—close to the 350 cells/μL global target for early detection—there were significant gaps among different demographic and geographic groups which warrant further attention. Our findings indicate some of the main contributing factors of HIV disparities in early detection as follows.

### 4.1. Regional Differences in HIV Early Detection

This is the first-of-its-kind study on the regional differences in early detection of HIV within Tajikistan and any country in Central Asia. Because of the deep cultural and political history of the broader region and its previous ties to the Soviet Union, the different economic, cultural, and social characteristics within the regions may contribute to disparities in access to HIV testing, stigma, and stage of detection.

Generally, compared to the capital city Dushanbe, Khatlon and DRS had earlier HIV detection while GBAO and Sughd suffered from later detection of PLHIV. Sughd and Khatlon had a greater improvement in early detection post-pandemic in 2021, but this gap with other regions narrowed in 2022 and 2023. This improvement may be due to additional financial support Sughd received from the U.S. Centers for Disease Control and Prevention (CDC) and the U.S. President’s Emergency Plan for AIDS Relief (PEPFAR) and Khatlon received from the Global Fund. Dushanbe was the only region to experience an improvement in detection from 2022 to 2023, while other regions are declining, likely due to shift of international financing to the capital. As the country’s capital, Dushanbe benefited from increased support from entities such as CDC/PEPFAR [54,55].

From 2010 to 2023, there were notable changes in the stage of HIV detection within and across regions. Median CD4 counts in Dushanbe were around 300 cells/μL in 2010, displaying fluctuations over time. Notable peaks were observed in 2013 (around 365 cells/μL) and 2021 (slightly below 400 cells/μL), stabilizing around 350 cells/μL by 2023 (see Figure 2). However, sociocultural factors such as education levels, discriminatory attitudes, and density of key populations including people who inject drugs (PWID), men who have sex with men (MSM), and commercial sex workers (CSWs) may still pose challenges to early detection efforts in Dushanbe [56].

GBAO, a large, predominantly rural, mountainous area in eastern Tajikistan bordering Afghanistan, stands as the nation’s poorest region and is inhabited by Tajikistan’s Ismaili Shia ethnic and religious minority [40]. Median CD4 counts in GBAO exhibited variability below 350 cells/μL throughout the study, with notable fluctuations particularly in 2010 (below 250 cells/μL) and 2021 (slightly below 400 cells/μL) (Figure 2). Despite its high education rates, GBAO faces challenges in HIV detection and testing coverage, indicating the need for targeted interventions to improve access to healthcare services in remote and underserved areas [56].

Sughd, situated in the northwest and contributing significantly to Tajikistan’s GDP, is made up of a predominantly Tajik ethnic population. Here, median CD4 counts fluctuated over subsequent years, peaking notably with the highest median CD4 of any region, a year after the onset of COVID-19, in 2021 (near 500 cells/μL), before declining to around 350 cells/μL by 2023 [Figure 2]. Sughd also experienced a significant improvement in early detection post-pandemic in 2021, but this gap narrowed in 2022 and 2023. These fluctuations in CD4 counts may reflect variations in access to healthcare services and awareness of HIV prevention methods within the region due to shifts in attention and resource allocation, potentially affecting initial efforts to address access gaps during the initial year of the COVID-19 pandemic.

Khatlon, located in the southwest with a majority Tajik population and Uzbeks as the second largest ethnic group, witnessed its median CD4 count starting at approximately 430 cells/μL in 2010, declining to around 250 cells/μL in 2020, and rebounding to slightly above 350 cells/μL by 2023. Khatlon demonstrated earlier detection compared to DRS and Dushanbe, suggesting relatively better access to HIV testing services. However, disparities in education levels and awareness of prevention methods persist, which may contribute to variations in HIV detection rates [56].

In the DRS, median CD4 counts commenced below 300 cells/μL in 2010, reaching a peak slightly above 400 cells/μL in 2013, and then remaining relatively stable within the range of 300–350 cells/μL until 2023 (Figure 2). Based on national survey data, this region exhibits the lowest rates of secondary education for both men and women, coupled with the lowest awareness of prevention methods and the highest prevalence of discriminatory attitudes towards PLHIV [56].

### 4.2. Age Disparities in HIV Early Detection

Our findings indicate that age has a significant impact on HIV early detection. Those under 19 years of age maintained significantly earlier HIV detection and a median CD4 count well above the 350 cells/μL global target compared to other age groups, for the most part, until 2021. In 2022 and 2023, however, there was a sharp decline in the median CD4 count, indicating barriers to early detection among those under 19 years of age post-pandemic. Those aged 19–39 years were consistently around or above the 350 cells/μL global target for early detection and the only age group with a sizable improvement post-COVID-19. Those above 39 years of age were consistently detected at a late stage. Individuals aged 39 and above consistently exhibited late-stage detection, placing them at a disadvantage compared to other age groups. Given their active engagement in age-related and sexual activities, they face a higher risk of HIV transmission if diagnosed late. Therefore, they require increased attention and targeted interventions.

As more vertically HIV-infected children survive and new horizontal HIV infections continue, the number of adolescents (aged 10–19 years) living with HIV is on the rise [57], although new HIV infections among older adolescents are decreasing [58]. While AIDS-related deaths are starting to decrease among younger adolescents, they are still on the rise among older adolescents [59]. Various risks and obstacles encountered during the stages of early (ages 10–14 years) and late (ages 15–19 years) adolescence can affect HIV-related outcomes and the likelihood of HIV infection and testing. Younger adolescents typically reside at home, are in the initial phases of puberty, and are less likely to have initiated sexual activity compared to their older counterparts. In late adolescence, gender disparities in HIV acquisition risk emerge, with older adolescent girls facing higher risks compared to boys [58]. Additional vulnerabilities arise for key adolescent populations, including young men who have sex with men (MSM), transgender individuals, commercial sex workers (CSW), and people who inject drugs (PWID) [60]. In Tajikistan, potential confounders may drive this earlier detection among those under 19 years of age: one, younger people when diagnosed are likely to have been infected for a shorter period of time, and two, Tajikistan has strong public health surveillance programs for the early diagnosis of perinatally infected persons (among pregnant mothers). Our findings support the conclusions of Tajikistan’s focused efforts to better reach young people who are at risk for HIV and detect them at earlier stages. Our findings further support prior literature which indicates that elderly individuals living with HIV are prone to delayed presentation [61].

### 4.3. Gender Variation in HIV Early Detection

Our findings further indicate that gender may be predictive of HIV early detection within certain time intervals. Throughout the time period, with the exception of 2020, females were detected around or above the 350 cells/μL global target while males were detected at a late stage every year, with the exception of 2021, which saw all-around increases for both genders.

The proportion of new HIV infections among women in Tajikistan has increased, rising from 31% in 2011 to 36% in 2022 [62]. Prior literature has demonstrated how women with HIV may face domestic violence, stigma, and discrimination [18,63,64,65]. While these may have impacts and cause potential barriers to testing and linkage to care, our findings align with prior literature which demonstrates women are more likely to test for HIV and seek care while men are disproportionately impacted by the stigma of HIV [66,67]. A similar study in the U.S. has shown women were more likely to be early diagnosed (with HIV having a higher baseline CD4 count at diagnosis) compared with men [68].

### 4.4. Area (Urban/Rural) Disparities in HIV Early Detection

Overall, the analysis reveals a fluctuating trend in median CD4 counts between rural and urban areas over the years, with rural populations generally exhibiting earlier detection compared to urban populations, except in 2015 and 2020. Notably, Tajikistan’s rural population percentage relative to the total population has ranged from 72% to 73% from 2010 to 2023, surpassing most nations, according to World Bank data [37].

Globally, several obstacles hinder rural residents from obtaining and utilizing dependable health information, including factors like geographical location, distance, adverse weather conditions, insufficient financial means, and limited availability of specialized healthcare services [69]. Studies indicate that rural dwellers typically possess lower socioeconomic status (SES) compared to urban counterparts, which could result in restricted accessibility to and utilization of health information owing to varying levels of access [70].

In urban areas with high population densities, individuals often enjoy anonymity, providing safe environments for key populations to connect. On the other hand, rural areas, characterized by smaller populations and close-knit communities, experience self-imposed social isolation as individuals must conceal their identities and have limited opportunities for covert networking [71,72].

While our findings contrast with global literature which concludes that rural populations face greater disparities in access to HIV primary care, testing, and other health care resources [73,74,75,76,77], our findings point to the importance of the types of key populations which may reside in urban areas compared to rural areas. Labor migrants are disproportionately more likely to reside in rural areas [78], including the wives of migrants who may be affected, while other key populations like PWID, MSM, and CSWs are more likely to reside in urban areas [65,79]. Our findings suggest Tajikistan has succeeded in ensuring access to health care in rural areas and potentially reaching migrant populations but may face increased difficulty detecting other key populations at an earlier stage.

In rural regions in Tajikistan, primary healthcare services are typically provided through medical houses, rural health centers, rural hospitals, and mobile clinics [80]. In contrast, urban areas offer a broader range of healthcare services, including primary and secondary care through polyclinics, family medicine centers, basic secondary care through district hospitals, and specialized secondary and intricate care in regional and national hospitals [80]. Moreover, urban areas tend to have a higher proportion of individuals with secondary education or higher compared to rural areas, which may influence health awareness and behaviors [80]. When considering HIV awareness and testing, urban areas exhibit greater awareness of prevention methods and self-test kits [56]. These multi-level characteristics of urban and rural populations in Tajikistan warrant further investigation.

### 4.5. Regional Disparities by Age in HIV Early Detection

Broadly, all regions follow the overall nationwide trend of earlier HIV detection among those under 19 and 19–39 years of age, and late detection among those above 39 years of age. However, there are specific time-specific changes which warrant attention. In 2015, Khatlon and GBAO experienced a significant decline to late detection among those under 19 years. In 2019, Sughd experienced a similar decline. Further, in 2022 and 2023 after the pandemic, DRS and Dushanbe saw a similar decline where those under 19 years of age were not only detected at a late stage, but later than all other age groups. The pandemic may have had some unintended consequences on detection of HIV among young people including those under 19 years of age which should be further investigated.

Other national health data from Tajikistan not related to HIV may provide additional insights into health disparities, thereby supplementing our findings. Based on a 2017 Demographic and Health Survey Atlas of Key Indicators, teenage childbearing (aged 15–19) is most common in DRS (9%) and least common in GBAO (2%) [56]. On the other hand, induced abortion varies by region, from 7% of women in GBAO to 13% of women in Sughd [56]. This lower rate of both teenage childbearing and abortions in GBAO compared to other regions may indicate the role of conservative cultural practices in GBAO, as documented in prior literature [40], which could justify GBAO’s improved detection for those under 19 years of age after COVID-19 while urban pockets like DRS and Dushanbe experienced later detection among those under 19 years of age. Similarly, most recent surveying indicates coverage of HIV counseling and testing during pregnancy is highest in GBAO (32%) and lowest in DRS (2%), which could explain earlier detection among those under 19 years of age from 2016 on in GBAO [56].

### 4.6. Regional Disparities by Gender in HIV Early Detection

Across regions, for the most part, males were disadvantaged and more likely to be detected at a late stage. However, in the capital Dushanbe, females were disadvantaged in certain years, including 2011, 2012, 2018, and 2020. Women may experience different barriers to early detection of HIV depending on the cultural, social, and public health characteristics of the region they reside in. To better understand the experience of PLHIV across regions, it is helpful to look at other population health disparities across regions. For example, women in Sughd are the most likely compared to other Tajikistan regions to receive at least four antenatal care (ANC) visits, have their births occur in health facilities, and have births assisted by a skilled provider (doctor, nurse, or midwife) [56]. This strong maternal healthcare, combined with increases in HIV testing, in Sughd may contribute to its continued earlier detection among females while explaining potential gender gaps in Dushanbe which can serve as barriers for women seeking HIV testing and care. Further, in Dushanbe, key populations like FSW may be driving later detection among females [39,40].

### 4.7. Regional Disparities by Area (Urban/Rural Status) in HIV Early Detection

Across all regions, the median CD4 counts in rural areas were often similar to or higher than those in urban areas for the same year. The stage of HIV detection among rural populations within Dushanbe varies due to its relatively small population size. Further, despite being primarily urban, the capital city, and the recipient of the most international funding, Dushanbe faces key barriers to HIV early detection compared to other regions, possibly related to various sociocultural factors such as education levels and discriminatory attitudes towards key populations, which warrant further examination. Examining the effects after COVID-19 starting in 2021, further investigation is needed to determine why rural populations in Sughd and DRS face a downward median CD4 count trajectory while Khatlon’s urban population faces a similar decline.

### 4.8. Limitations

There are a few limitations to consider in this study. Primarily, we utilized cross-sectional data from the Tajikistan Ministry of Health and Social Protection which only includes clinically-diagnosed cases by the Tajikistani health system and may not include potential HIV cases or deaths, particularly among key or stigmatized populations. Some regions may have underreported or overreported data. Secondly, our primary dependent variable used for findings is median CD4 at time of HIV detection and there may be other measures that should be considered. Thirdly, we calculated the median CD4 count for each calendar year, which is visualized in our figures; however, our statistical models treat time as continuous, which allows for more specific predictive analysis at the month and day level. Fourthly, we included 2020 HIV data which is adversely affected by the start of the COVID-19 pandemic. Finally, our characterization of “urban/rural” status is based upon the Tajikistan Ministry of Health and Social Protection definition in health centers and may not reflect full population demographics.

## 5. Conclusions

Looking at the national HIV data of Tajikistan across all years from 2010 to 2023, our overall findings indicate that region and age are significant predictors of the stage of HIV detection. Gender and urban/rural status may be predictors in specific time periods. The Khatlon region serves as a case study for best practices in early detection of HIV. There appears to be a notable gap concerning age and gender in HIV detection. Individuals above 39 years of age and males are predominantly detected at a late stage, suggesting a need for further attention and targeted interventions in these demographic groups. The onset of COVID-19 initially led to a decline in early HIV detection. However, early detection was accelerated in 2021. Despite this, most regions have returned to near pre-pandemic levels of detection, underscoring the importance of sustainable financing in maintaining effective HIV detection efforts. More research is needed to identify what root causes drive the differential stage of HIV detection and testing uptake among different regions, age groups, genders, and areas. Disparities in early HIV detection contribute to differences in the burden of disease experienced by specific populations. These disparities are of public health significance in order to address UNAIDS’ “95–95–95” targets [81] to effectively diagnose PLHIV, provide antiretroviral therapy (ART), and achieve viral suppression.

## Figures and Tables

**Table 1:** Demographic characteristics of HIV cases in Tajikistan from 2010 to 2023 (N = 10,700).

Variable	Count (%)
Region of Residence	
Districts of Republican Subordination (DRS)	2207 (20.63)
Dushanbe	2640 (24.67)
Gorno-Badakhshan Autonomous Oblast (GBAO)	363 (3.39)
Khatlon	3047 (28.48)
Sughd	2410 (22.52)
No Response	33 (0.31)
Age Group	
<19 y	1326 (12.39)
19–39 y	6629 (61.95)
>39 y	2745 (25.65)
Gender	
Female	4516 (42.21)
Male	6184 (57.79)
Area of Residence	
Rural	5999 (56.07)
Urban	4667 (43.62)
No Response	34 (0.32)

**Table 2:** Median regression models with median CD4 cell count outcome.

Variable	Model 1	Model 2	Model 3
	Coef. (SE); *p*-Value	Coef. (SE); *p*-Value	Coef. (SE); *p*-Value
**Intercept**	319.11 (7.59); <0.001	298.03 (13.5); <0.001	305.08 (8.33); <0.001
**Years (since 2010)**	3.56 (0.89); <0.001	3.92 (1.77); 0.027	7.75 (1.03); <0.001
**Region of Residence**	—		—
Dushanbe		Reference *	
Districts of Republican Subordination		41.46 (20.75); 0.046	
Gorno-Badakhshan Autonomous Oblast		−48.3 (28.61); 0.091	
Khatlon		76.52 (21.48); <0.001	
Sughd		−10.73 (22.46); 0.633	
**Age Group**	—	—	
19–39 y			Reference *
<19 y			311.68 (44.09); <0.001
>39 y			−61.24 (14.63); <0.001
**Interaction:**	—		—
**Years (since 2010) × Region of Residence**			
Dushanbe		Reference *	
Districts of Republican Subordination		−5.06 (2.53); 0.046	
Gorno-Badakhshan Autonomous Oblast		1.57 (4.58); 0.732	
Khatlon		−3.09 (2.53); 0.223	
Sughd		4.44 (2.67); 0.096	
**Years (since 2010) × Age Group:**	—	—	
19–39 y			Reference *
<19 y			−29.68 (4.93); <0.001
>39 y			−4.22 (1.78); 0.018

Notes: Model 1: Time (years since 2010) as independent variable. Model 2: Time (years since 2010) and region of residence as independent variables along with the two-way interactions between time and region. Wald test *p*-value for interaction between time and region: *p* = 0.006. Model 3: Time (years since 2010) and age as independent variables along with the two-way interactions between time and age. Wald test *p*-value for interaction between time and age: *p* < 0.001. * The reference group’s median CD4 cell count values were calculated using the coefficient values for intercept and years (since 2010).

## Data Availability

The data that support the findings of this study are the property of the Ministry of Health of Tajikistan and are not publicly available.

## References

[1] Poorolajal J., Hooshmand E., Mahjub H., Esmailnasab N., Jenabi E. (2016). Survival Rate of AIDS Disease and Mortality in HIV-Infected Patients: A Meta-Analysis. Public Health.

[2] Hammond R., Harry T.C. (2008). Efficacy of Antiretroviral Therapy in Africa: Effect on Immunological and Virological Outcome Measures—A Meta-Analysis. Int. J. STD AIDS.

[3] Braunstein S.L., Robertson M.M., Myers J., Nash D. (2016). Using HIV Viral Load from Surveillance to Estimate the Timing of Antiretroviral Therapy Initiation. J. Acquir. Immune Defic. Syndr..

[4] Ding Y., Duan S., Wu Z., Ye R., Yang Y., Yao S., Wang J., Xiang L., Jiang Y., Lu L. (2016). Timing of Antiretroviral Therapy Initiation after Diagnosis of Recent Human Immunodeficiency Virus Infection and CD4(+) T-Cell Recovery. Clin. Microbiol. Infect.

[5] May M.T., Gompels M., Delpech V., Porter K., Orkin C., Kegg S., Hay P., Johnson M., Palfreeman A., Gilson R. (2014). Impact on Life Expectancy of HIV-1 Positive Individuals of CD4+ Cell Count and Viral Load Response to Antiretroviral Therapy. AIDS.

[6] HIV—Global. https://www.who.int/health-topics/hiv-aids.

[7] Antinori A., Coenen T., Costagiola D., Dedes N., Ellefson M., Gatell J., Girardi E., Johnson M., Kirk O., Lundgren J. (2011). Late Presentation of HIV Infection: A Consensus Definition. HIV Med..

[8] Technical Notes|Volume 26 Number 2|HIV Surveillance|Reports|Resource Library|HIV/AIDS|CDC. https://www.cdc.gov/hiv/library/reports/hiv-surveillance/vol-26-no-2/content/technical-notes.html.

[9] Gueler A., Moser A., Calmy A., Günthard H.F., Bernasconi E., Furrer H., Fux C.A., Battegay M., Cavassini M., Vernazza P. (2017). Life Expectancy in HIV-Positive Persons in Switzerland: Matched Comparison with General Population. AIDS.

[10] Marcus J.L., Chao C.R., Leyden W.A., Xu L., Charles P., Quesenberry J., Klein D.B., Towner W.J., Horberg M.A., Silverberg M.J. (2016). Narrowing the Gap in Life Expectancy Between HIV-Infected and HIV-Uninfected Individuals With Access to Care. J. Acquir. Immune Defic. Syndr..

[11] Edwards J.K., Cole S.R., Breger T.L., Rudolph J.E., Filiatreau L.M., Buchacz K., Humes E., Rebeiro P.F., D’Souza G., Gill M.J. (2021). Mortality among People Entering HIV Care Compared to the General US Population: An Observational Study. Ann. Intern. Med..

[12] Lewden C., Bouteloup V., De Wit S., Sabin C., Mocroft A., Wasmuth J.C., van Sighem A., Kirk O., Obel N., Collaboration of Observational HIV Epidemiological Research Europe (COHERE) in EuroCoord (2012). All-Cause Mortality in Treated HIV-Infected Adults with CD4 ≥ 500/mm^3^ Compared with the General Population: Evidence from a Large European Observational Cohort Collaboration. Int. J. Epidemiol..

[13] van Lelyveld S.F.L., Gras L., Kesselring A., Zhang S., De Wolf F., Wensing A.M.J., Hoepelman A.I.M., ATHENA national observational cohort study (2012). Long-Term Complications in Patients with Poor Immunological Recovery despite Virological Successful HAART in Dutch ATHENA Cohort. AIDS.

[14] Mocroft A., Furrer H.J., Miro J.M., Reiss P., Mussini C., Kirk O., Abgrall S., Ayayi S., Bartmeyer B., Braun D. (2013). The Incidence of AIDS-Defining Illnesses at a Current CD4 Count ≥ 200 Cells/μL in the Post-Combination Antiretroviral Therapy Era. Clin. Infect. Dis..

[15] Mocroft A., Laut K., Reiss P., Gatell J., Ormaasen V., Cavassini M., Hadziosmanovic V., Mansinho K., Pradier C., Vasylyev M. (2018). Where Is the Greatest Impact of Uncontrolled HIV Infection on AIDS and Non-AIDS Events in HIV?. AIDS.

[16] Guaraldi G., Zona S., Menozzi M., Brothers T.D., Carli F., Stentarelli C., Dolci G., Santoro A., Da Silva A.R.D., Rossi E. (2017). Late Presentation Increases Risk and Costs of Non-Infectious Comorbidities in People with HIV: An Italian Cost Impact Study. AIDS Res. Ther..

[17] Martin-Iguacel R., Reyes-Urueña J., Bruguera A., Aceitón J., Díaz Y., Moreno-Fornés S., Domingo P., Burgos-Cibrian J., Tiraboschi J.M., Johansen I.S. (2022). Determinants of Long-Term Survival in Late HIV Presenters: The Prospective PISCIS Cohort Study. eClinicalMedicine.

[18] Eastern Europe and Central Asia May Face an Accelerated Increase in New HIV Infections and AIDS-Related Deaths Because of the Humanitarian Crisis Gripping the Entire Region. https://www.unaids.org/en/resources/presscentre/featurestories/2022/october/20221027_eastern-europe-central-asia.

[19] Licata F., Angelillo S., Nobile C.G.A., Di Gennaro G., Bianco A. (2022). Understanding Individual Barriers to HIV Testing Among Undergraduate University Students: Results From a Cross-Sectional Study in Italy. Front. Med..

[20] Peltzer K., Matseke G. (2013). Determinants of HIV Testing among Young People Aged 18–24 Years in South Africa. Afr. Health Sci..

[21] Gebregziabher M., Dai L., Vrana-Diaz C., Teklehaimanot A., Sweat M. (2018). Gender Disparities in Receipt of HIV Testing Results in Six Sub-Saharan African Countries. Health Equity.

[22] Obermeyer C.M., Osborn M. (2007). The Utilization of Testing and Counseling for HIV: A Review of the Social and Behavioral Evidence. Am. J. Public Health.

[23] Genberg B.L., Hlavka Z., Konda K.A., Maman S., Chariyalertsak S., Chingono A., Mbwambo J., Modiba P., Van Rooyen H., Celentano D.D. (2009). A Comparison of HIV/AIDS-Related Stigma in Four Countries: Negative Attitudes and Perceived Acts of Discrimination towards People Living with HIV/AIDS. Soc. Sci. Med..

[24] Assefa Y., Van Damme W., Mariam D.H., Kloos H. (2010). Toward Universal Access to HIV Counseling and Testing and Antiretroviral Treatment in Ethiopia: Looking beyond HIV Testing and ART Initiation. AIDS Patient Care STDS.

[25] Musheke M., Ntalasha H., Gari S., McKenzie O., Bond V., Martin-Hilber A., Merten S. (2013). A Systematic Review of Qualitative Findings on Factors Enabling and Deterring Uptake of HIV Testing in Sub-Saharan Africa. BMC Public Health.

[26] Kasymova S. (2020). Uptake of HIV Testing among Women of Reproductive Age in Tajikistan: An Assessment of Individual Determinants. Cent. Asian J. Glob. Health.

[27] Nash D., Katyal M., Shah S. (2005). Trends in Predictors of Death Due to HIV-Related Causes among Persons Living with AIDS in New York City: 1993–2001. J. Urban Health.

[28] Giordano T.P., Gifford A.L., White A.C., Suarez-Almazor M.E., Rabeneck L., Hartman C., Backus L.I., Mole L.A., Morgan R.O. (2007). Retention in Care: A Challenge to Survival with HIV Infection. Clin. Infect. Dis..

[29] Nelson J.A., Kinder A., Johnson A.S., Hall H.I., Hu X., Sweet D., Guido A., Katner H., Janelle J., Gonzalez M. (2018). Differences in Selected HIV Care Continuum Outcomes Among People Residing in Rural, Urban, and Metropolitan Areas-28 US Jurisdictions. J. Rural. Health.

[30] Cities Ending the AIDS Epidemic. https://www.unaids.org/sites/default/files/media_asset/cities-ending-the-aids-epidemic_en.pdf.

[31] Magadi M.A. (2017). Understanding the Urban-Rural Disparity in HIV and Poverty Nexus: The Case of Kenya. J. Public Health.

[32] Weissman S., Duffus W.A., Iyer M., Chakraborty H., Samantapudi A.V., Albrecht H. (2015). Rural-Urban Differences in HIV Viral Loads and Progression to AIDS among New HIV Cases. South Med. J..

[33] He L., Pan X., Yang J., Ma Q., Jiang J., Wang W., Qiu J., Zou Y., Wang P., Zhao D. (2020). HIV Risk Behavior and HIV Testing among Rural and Urban Men Who Have Sex with Men in Zhejiang Province, China: A Respondent-Driven Sampling Study. PLoS ONE.

[34] Preston D.B., D’Augelli A.R., Kassab C.D., Cain R.E., Schulze F.W., Starks M.T. (2004). The Influence of Stigma on the Sexual Risk Behavior of Rural Men Who Have Sex with Men. AIDS Educ. Prev..

[35] Ohl M.E., Perencevich E. (2011). Frequency of Human Immunodeficiency Virus (HIV) Testing in Urban vs. Rural Areas of the United States: Results from a Nationally-Representative Sample. BMC Public Health.

[36] Cope A.B., Powers K.A., Serre M.L., Escamilla V., Emch M.E., Leone P.A., Mobley V.L., Miller W.C. (2016). Distance to Testing Sites and Its Association with Timing of HIV Diagnosis. AIDS Care.

[37] World Bank Open Data. https://data.worldbank.org.

[38] Nations U. HIV Epidemic Control in Central Asia Still Has a Long Way to Go. https://www.un.org/en/un-chronicle/hiv-epidemic-control-central-asia-still-has-long-way-go.

[39] King E.J., Maksymenko K.M., Almodovar-Diaz Y., Johnson S. (2016). “If She Is a Good Woman…” and “to Be a Real Man…”: Gender, Risk and Access to HIV Services among Key Populations in Tajikistan. Cult. Health Sex.

[40] Alaei A., Bromberg D.J., Tate M.M., Karimov S., Saidi D., Alaei K. (2021). HIV and Associated Factors among Female Sex Workers in Tajikistan: Analysis from a National Bio-Behavioral Survey. Int. J. STD AIDS.

[41] Ibragimov U., Wong F.Y. (2018). Qualitative Examination of Enacted Stigma towards Gay and Bisexual Men and Related Health Outcomes in Tajikistan, Central Asia. Glob. Public Health.

[42] Ancker S., Rechel B. (2015). Policy Responses to HIV/AIDS in Central Asia. Glob. Public Health.

[43] Central Asia and the War in Ukraine. https://www.hoover.org/research/central-asia-and-war-ukraine.

[44] Murakami E. (2022). Immediate Impacts of the COVID-19 Pandemic on Household Economic Activities and Food Security in Tajikistan. Econ. Disaster Clim. Change.

[45] Yamada E., Shimizutani S. (2022). The COVID 19 Pandemic, Daily Mobility and Household Welfare: Evidence from Tajikistan. Transp. Res. Interdiscip. Perspect..

[46] Birungi C., Haacker M., Taramusi I., Mpofu A., Madzima B., Apollo T., Mugurungi O., Odiit M., Obst M.A. (2022). Economic Implications of COVID-19 for the HIV Epidemic and the Response in Zimbabwe. Afr. J. AIDS Res..

[47] Chenneville T., Gabbidon K., Hanson P., Holyfield C. (2020). The Impact of COVID-19 on HIV Treatment and Research: A Call to Action. Int. J. Environ. Res. Public Health.

[48] IN DANGER: UNAIDS Global AIDS Update 2022. https://www.unaids.org/sites/default/files/media_asset/2022-global-aids-update_en.pdf.

[49] Alaei A., Nautiyal N., Mishkin K., Saifuddin Karimov D., Saidi D., Alaei K. (2018). Factors Associated with Linkage to Care for HIV Patients in Tajikistan. Int. J. STD AIDS.

[50] Cantelmo C.B., Lee B., Dutta A. (2019). Smart Cascades: Using Cost Analysis to Improve HIV Care and Treatment Interventions to Achieve Global 95-95-95 Goals. Afr. J. AIDS Res..

[51] Bassett G., Koenker R. (1982). Tests of Linear Hypotheses and L1 Estimation. Econometrica.

[52] R Core Team (2023). R: A Language and Environment for Statistical Computing. R Foundation for Statistical Computing, Vienna, Austria. https://www.R-project.org/.

[53] Gatechompol S., Avihingsanon A., Putcharoen O., Ruxrungtham K., Kuritzkes D.R. (2021). COVID-19 and HIV Infection Co-Pandemics and Their Impact: A Review of the Literature. AIDS Res. Ther..

[54] U.S. Embassy in Tajikistan https://tj.usembassy.gov/pepfar-and-usaid-launch-new-program-in-tajikistan-to-combat-hiv-aids/.

[55] Chun H.M., Dirlikov E., Cox M.H., Sherlock M.W., Obeng-Aduasare Y., Sato K., Voetsch A.C., Ater A.D., Romano E.R., Tomlinson H. (2023). Vital Signs: Progress toward Eliminating HIV as a Global Public Health Threat Through Scale-Up of Antiretroviral Therapy and Health System Strengthening Supported by the U.S. President’s Emergency Plan for AIDS Relief—Worldwide, 2004–2022. MMWR Morb. Mortal. Wkly. Rep..

[56] Statistical Agency under the President of the Republic of Tajikistan, Ministry of Health and Social Protection of Population of the Republic of Tajikistan, and ICF (2018). Tajikistan Demographic and Health Survey 2017.

[57] Slogrove A.L., Mahy M., Armstrong A., Davies M.-A. (2017). Living and Dying to Be Counted: What We Know about the Epidemiology of the Global Adolescent HIV Epidemic. J. Int. AIDS Soc..

[58] Young People and HIV. https://www.unaids.org/sites/default/files/media_asset/young-people-and-hiv_en.pdf.

[59] AIDSinfo|UNAIDS. https://aidsinfo.unaids.org/.

[60] Bekker L.-G., Johnson L., Wallace M., Hosek S. (2015). Building Our Youth for the Future. J. Int. AIDS Soc..

[61] Smith R.D., Delpech V.C., Brown A.E., Rice B.D. (2010). HIV Transmission and High Rates of Late Diagnoses among Adults Aged 50 Years and Over. AIDS.

[62] Tajikistan Takes a Positive Step towards Decriminalization of HIV Exposure and Transmission. https://www.unaids.org/en/resources/presscentre/featurestories/2024/january/300124_Tajikistan_decrim_steps_hiv_exposure.

[63] Thorne C., Ferencic N., Malyuta R., Mimica J., Niemiec T. (2010). Central Asia: Hotspot in the Worldwide HIV Epidemic. Lancet Infect Dis.

[64] HIV Programme Review in Tajikistan. https://www.chip.dk/Portals/0/files/CC%20WHO/HIV-Programme-Review-in-Tajikistan%20(final%20report).pdf.

[65] Smolak A., El-Bassel N. (2013). Multilevel Stigma as a Barrier to HIV Testing in Central Asia: A Context Quantified. AIDS Behav..

[66] Women Are More Likely to Be on HIV Treatment. https://www.unaids.org/en/resources/presscentre/featurestories/2020/april/20200428_women-more-likely-to-be-on-hiv-treatment.

[67] Ha J.H., Lith L.M.V., Mallalieu E.C., Chidassicua J., Pinho M.D., Devos P., Wirtz A.L. (2019). Gendered Relationship between HIV Stigma and HIV Testing among Men and Women in Mozambique: A Cross-Sectional Study to Inform a Stigma Reduction and Male-Targeted HIV Testing Intervention. BMJ Open.

[68] Almirol E.A., McNulty M.C., Schmitt J., Eavou R., Taylor M., Tobin A., Ramirez K., Glick N., Stamos M., Schuette S. (2018). Gender Differences in HIV Testing, Diagnosis, and Linkage to Care in Healthcare Settings: Identifying African American Women with HIV in Chicago. AIDS Patient Care STDS.

[69] McIlhenny C.V., Guzic B.L., Knee D.R., Wendekier C.M., Demuth B.R., Roberts J.B. (2011). Using Technology to Deliver Healthcare Education to Rural Patients. Rural. Remote Health.

[70] The 2014 Update of the Rural-Urban Chartbook. https://ruralhealth.und.edu/projects/health-reform-policy-research-center/pdf/2014-rural-urban-chartbook-update.pdf.

[71] (2002). Who Am I in Relation to Them?: Gay, Lesbian, and Queer People Leave the City to Attend Rural Family Weddings—RAMONA FAITH OSWALD. https://journals.sagepub.com/doi/abs/10.1177/0192513x02023003001.

[72] D’Augelli A.R., Hart M.M. (1987). Gay women, men, and families in rural settings: Toward the development of helping communities. Am. J. Community Psychol..

[73] Bono R.S., Pan Z., Dahman B., Deng Y., Kimmel A.D. (2023). Urban-Rural Disparities in Geographic Accessibility to Care for People Living with HIV. AIDS Care.

[74] Dey N.E.Y., Owusu Ansah K., Norman Q.A., Manukure J.M., Brew A.B.K., Dey E.A., Agbadi P. (2022). HIV Testing among Sexually Active Ghanaians: An Examination of the Rural-Urban Correlates. AIDS Behav..

[75] Maulide Cane R., Melesse D.Y., Kayeyi N., Manu A., Wado Y.D., Barros A., Boerma T. (2021). HIV Trends and Disparities by Gender and Urban-Rural Residence among Adolescents in Sub-Saharan Africa. Reprod. Health.

[76] Zhang L., Chow E.P.F., Jahn H.J., Kraemer A., Wilson D.P. (2013). High HIV Prevalence and Risk of Infection among Rural-to-Urban Migrants in Various Migration Stages in China: A Systematic Review and Meta-Analysis. Sex Transm. Dis..

[77] He N. (2007). Sociodemographic Characteristics, Sexual Behavior, and HIV Risks of Rural-to-Urban Migrants in China. Biosci. Trends.

[78] Strengthening Support for Labor Migration in Tajikistan. https://www.adb.org/sites/default/files/publication/656481/support-labor-migration-tajikistan.pdf.

[79] Beyrer C., Patel Z., Stachowiak J.A., Tishkova F.K., Stibich M.A., Eyzaguirre L.M., Carr J.K., Mogilnii V., Peryshkina A., Latypov A. (2009). Characterization of the Emerging HIV Type 1 and HCV Epidemics among Injecting Drug Users in Dushanbe, Tajikistan. AIDS Res. Hum. Retroviruses.

[80] Khodjamurodov G., Rechel B. (2010). Tajikistan: Health System Review. Health Syst. Transit..

[81] Global AIDS Strategy 2021–2026. https://www.unaids.org/en/Global-AIDS-Strategy-2021-2026.

